# Nasopharyngeal carriage and risk factors of major meningitis pathogens among asymptomatic healthcare workers in paediatric units in Benin, with serogroup distribution of *Neisseria meningitidis*

**DOI:** 10.1186/s12879-025-11492-3

**Published:** 2025-08-22

**Authors:** Chakir Ishola Bello, Cyriaque Comlan Degbey, Yves Eric Denon, Sessi Armel R. Hinvi, Lamine Baba-Moussa

**Affiliations:** 1 Laboratory of Biology and Molecular Typing in Microbiology, Faculty of Sciences and Technology, University of Abomey-Calavi, P.O. Box 1604, Cotonou, 01 BP 188 Benin; 2 Microbiology Laboratory, Mono Departmental Hospital Center, P. O. Box 62, Lokossa, Benin; 3 Department of Environmental Health, Regional Institute of Public Health, University of Abomey-Calavi, Ouidah, Benin; 4 Littoral Department, University Hospital Hygiene Clinic, Hubert Koutoukou Maga National Teaching Hospital (CNHU-HKM), Cotonou, Benin; 5 Diagnostic and Exploratory Department, Ministry of Health of Benin, P. O. Box 882, Cotonou, Benin

**Keywords:** Nasopharyngeal, Healthcare worker, Asymptomatic carriage, Paediatric units

## Abstract

**Background:**

Healthcare workers in paediatric settings are in close contact with vulnerable children and may serve as reservoirs and vectors for the transmission of respiratory pathogens such as *Streptococcus pneumoniae*, *Neisseria meningitidis*, and *Haemophilus influenzae*. In the African meningitis belt, data on asymptomatic carriage of these pathogens among healthcare workers remain scarce. This study aimed to assess the nasopharyngeal carriage of these three major meningitis-associated bacteria among paediatric healthcare personnel in Benin.

**Methods:**

A cross-sectional study was conducted from September 2023 to December 2024 among 221 healthcare workers working in paediatric and neonatology units. Nasopharyngeal swabs were collected and analysed using quantitative real-time Polymerase Chain Reaction to detect *S. pneumoniae* (lytA), *N. meningitidis* (sodC), and *H. influenzae* (hpd3), as well as to characterise *N. meningitidis* serogroups and detect *H. influenzae* type b. Sociodemographic and clinical data were collected through a structured questionnaire. Associations between participant characteristics and pathogen carriage were assessed using chi-square tests and binary logistic regression, with a significance threshold set at *p* < 0.05.

**Results:**

The nasopharyngeal carriage prevalence was 24.4% (54/221) for *S. pneumoniae*, 11.8% (26/221) for *H. influenzae*, and 10.0% (22/221) for *N. meningitidis*. Among the meningococcal carriers, 8 (36.4%) carried serogroup Y, 3 (13.6%) serogroup X, and 1 (4.5%) serogroup A. The remaining positive samples did not belong to any of the major vaccine-targeted serogroups (A, B, C, W, X, Y). None of the *Haemophilus influenzae*-positive samples were of serotype b. Pneumococcal carriage was significantly associated with younger age (18–35 years), and residence within the meningitis belt (*p* < 0.05). Similarly, *Neisseria meningitidis* carriage showed a significant association with residence in the meningitis belt. In contrast, no significant association was observed between *Haemophilus influenzae* carriage and any of the variables assessed.

**Conclusion:**

Our findings reveal a substantial carriage of meningitis-related pathogens among paediatric healthcare workers, notably *Streptococcus pneumoniae*,* Haemophilus influenzae* and *Neisseria meningitidis*. The predominance of non-vaccine *N. meningitidis* serogroups (Y and X) and the absence of *H. influenzae* b suggest the need for expanded molecular surveillance and reconsideration of vaccine strategies targeting healthcare workers. These results emphasize the importance of infection prevention and control measures measures in paediatric care settings.

**Supplementary Information:**

The online version contains supplementary material available at 10.1186/s12879-025-11492-3.

## Introduction

Invasive bacterial infections, notably meningitis caused by *Streptococcus pneumoniae*, *Neisseria meningitidis*, and *Haemophilus influenzae*, remain a major public health concern, especially in resource-limited settings [1, 2]. Beyond meningitis, these pathogens also cause severe pneumonia and sepsis, particularly in children and immunocompromised individuals [3]. Although global vaccination programmes have contributed to a significant reduction in the incidence of these infections, asymptomatic nasopharyngeal carriage continues to play a crucial role in transmission dynamics, particularly in high-risk environments such as healthcare facilities [1]. Nasopharyngeal carriage is typically asymptomatic and age-related, with peak prevalence often observed during adolescence [4]. However, asymptomatic carriers serve as silent reservoirs, enabling the horizontal spread of these pathogens both in the community and within healthcare setting [3]. This is particularly concerning in paediatric wards, where patients are highly vulnerable to severe infections. Notably, the carriage of *S. pneumoniae*, *N. meningitidis*, and *H. influenzae* is a key precursor to invasive disease, especially meningitis [3, 4]. In this context, healthcare workers (HCWs), by virtue of their contact with both patients and the community, may act as important vectors in the nosocomial transmission of these bacteria. Surveillance of bacterial carriage among HCWs is therefore essential to better understand transmission dynamics, implement appropriate infection prevention and control (IPC) strategies, and inform vaccination policies [5, 6].

In sub-Saharan Africa, data on asymptomatic bacterial carriage among HCWs remain limited. According to 2019 health statistics from Benin, acute respiratory infections were the second leading cause of medical consultation among children aged 0 to 5 years (17%), following malaria (48.8%) [7]. This high burden of respiratory illnesses likely sustains continuous exposure of paediatric healthcare workers to respiratory pathogens. Moreover, *S. pneumoniae*, *N. meningitidis*, and *H. influenzae* are among the pathogens most frequently isolated from cerebrospinal fluid samples [8], highlighting the occupational exposure risk for staff in paediatric and neonatology units. In Benin, the National Agency for Primary Health Care is responsible for implementing the Expanded Programme on Immunization (EPI). As part of the routine immunisation schedule, children receive the *Haemophilus influenzae* type b (Hib) vaccine and the 13-valent pneumococcal conjugate vaccine (PCV13) at 6, 10, and 14 weeks of age. Since 2021, the meningococcal serogroup A conjugate vaccine (MenA) has also been included in the national immunisation schedule and is administered at 9 months of age. However, data on national coverage rates for meningococcal vaccination are not publicly available. There is also no national vaccination programme targeting adults or healthcare workers (HCWs) specifically. Outside of mass vaccination campaigns conducted during meningococcal outbreaks, primarily targeting serogroups A or C, vaccination of HCWs is neither systematically implemented nor subsidised. As a result, most HCWs remain unvaccinated against *N. meningitidis*, *S. pneumoniae*, and *H. influenzae*, despite their increased risk of exposure. Assessing the prevalence, serotype or serogroup distribution, and associated risk factors of these bacteria among healthcare workers is essential to inform immunisation strategies and enhance infection prevention and control measures, especially given the limited availability of vaccines against certain meningococcal serogroups.

This study aimed to determine the prevalence, molecular characteristics, and risk factors associated with asymptomatic nasopharyngeal carriage of *Streptococcus pneumoniae*, *Haemophilus influenzae*, and *Neisseria meningitidis* among healthcare personnel working in paediatric units in Benin, thereby addressing critical knowledge gaps to inform targeted interventions.

## Methods

### Type of study

This observational cross-sectional study was conducted from September 2023 to December 2024 in the paediatric and neonatology units of selected healthcare facilities in Benin. The study aimed to determine the prevalence, molecular characteristics, and associated risk factors of asymptomatic nasopharyngeal carriage of *Streptococcus pneumoniae*, *Haemophilus influenzae*, and *Neisseria meningitidis* among healthcare personnel.

**Fig. 1:**
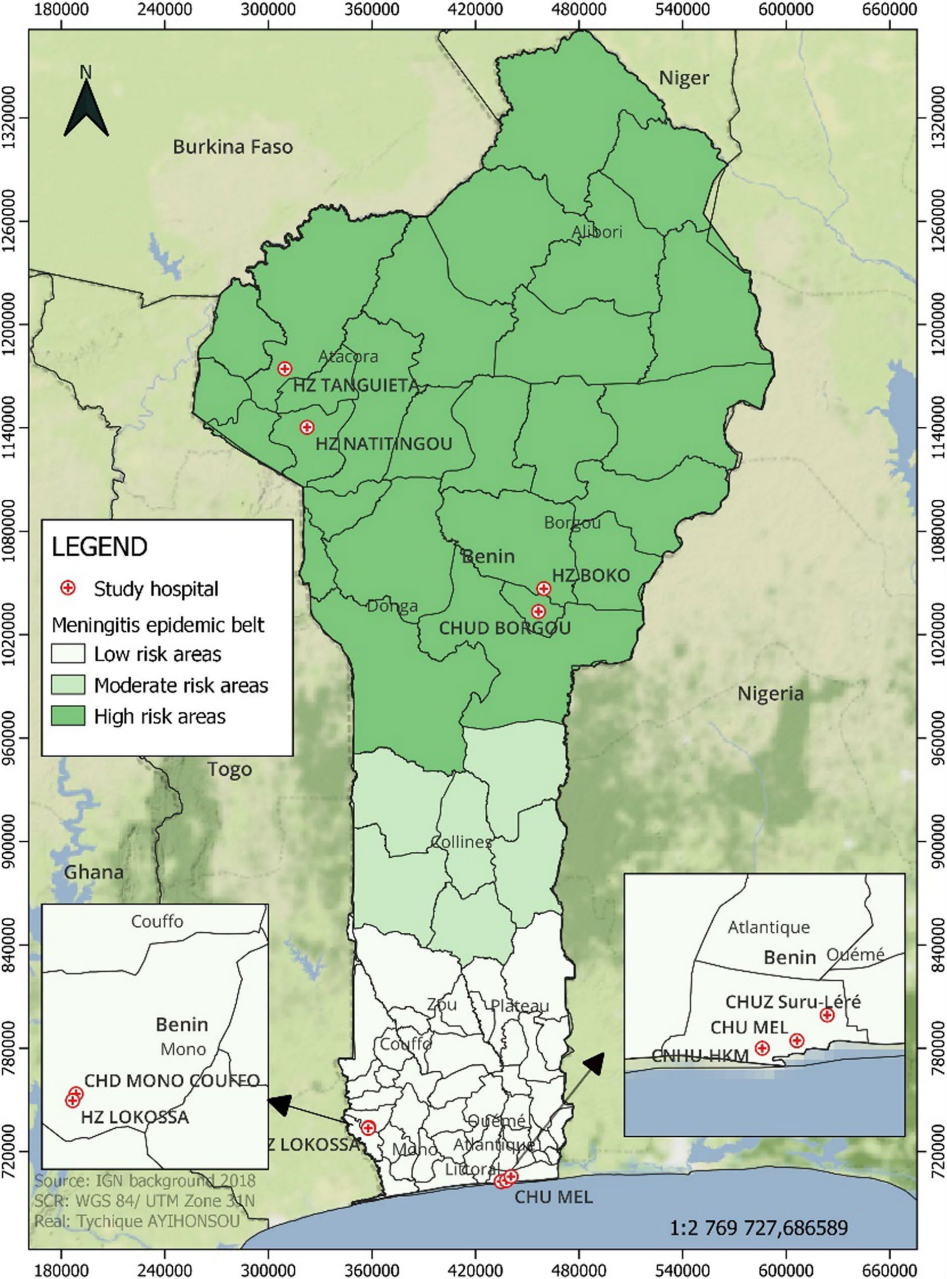
Map of the study area locations and regions within and outside the African meningitis belt

Benin, a West African country, lies partially within the African meningitis belt, with approximately half of the study sites located within this zone, particularly in the northern regions. The remaining sites, situated in the southern part of the country, are located outside or at the edge of this epidemiological zone. The participating hospitals were situated in both urban and peri-urban settings and provided care to a large paediatric population. For geographic context, Fig. 1 presents a map of Benin, highlighting the study locations and the limits of the meningitis belt.

### Study population and inclusion criteria

The study population consisted of healthcare workers (HCWs) directly involved in the care of children, including paediatricians, general practitioners, nurses, and nursing assistants. In our setting, due to a shortage of personnel, staff working in the neonatology units also routinely provide care to older infants and children in general paediatric wards. Therefore, their inclusion reflects regular exposure to age groups with higher nasopharyngeal colonisation rates. Eligible participants met the following inclusion criteria: (i) active employment in paediatric or neonatology wards for at least one month prior to sampling, (ii) absence of respiratory symptoms at the time of sample collection, and (iii) provision of written informed consent. HCWs who had received antibiotic treatment within the 14 days preceding sample collection were excluded to minimise the risk of false-negative carriage results due to recent antimicrobial exposure.

### Sample size estimation

The sample size was estimated using Cochran’s formula for single population proportion:

N = (𝑍²_𝛼_×𝑃×𝑄)/i², where P is the estimated prevalence, i the desired margin of error, and 𝑍_𝛼_​ the Z-score for a 95% confidence interval. Based on previous data from the African meningitis belt, the adult carriage prevalence of *S. pneumoniae* was estimated at 22% [9]. Using a 6% margin of error and a 95% confidence interval, the calculated target sample size was 183. A total of 221 HCWs (approximately 121% of target) were enrolled consecutively, which enhanced the statistical power and reliability of the study findings.

### Data and specimen collection

Before sample collection, all eligible HCWs received detailed verbal and written information about the study and provided written informed consent. Each participant completed a structured questionnaire designed to collect sociodemographic data, recent respiratory symptoms, occupational exposure, and potential risk factors for bacterial carriage such as vaccination status, cohabitation with young children, and residence in high-risk areas for meningitis. This questionnaire was specifically developed for the present study (see Supplementary File 1) and was pilot-tested for clarity and relevance prior to its implementation.

Nasopharyngeal swabs were collected using sterile nylon-flocked swabs (Shenzhen KangDaAn Biotechnology Co., China) following WHO Pneumococcal Carriage Working Group updated guidelines [10]. Swabs were immediately placed in Amies transport medium, kept between 2 and 8 °C, and transported promptly to the Laboratory of Biology and Molecular Typing in Microbiology, University of Abomey-Calavi. Upon arrival, samples were vortexed, aliquoted (200 µL), and stored at − 20 °C pending analysis, ensuring nucleic acid integrity for molecular testing.

### Molecular analyses

Real-time PCR assays were performed using the AriaMx™ Real-Time PCR system (Agilent Technologies) employing previously validated primers and hydrolysis probes targeting the *lytA* gene for *S. pneumoniae*, *hpd3* gene for *H. influenzae*, and *sodC* gene for *N. meningitidis*. A cycle threshold (Ct) value below 36 was considered indicative of a positive result, in line with established diagnostic criteria. Each PCR run included no-template negative controls and positive controls to ensure assay reliability and quality control. For *N. meningitidis*-positive samples, capsular serogrouping was carried out using serogroup-specific primers targeting A, B, C, W, X, and Y. For *H. influenzae*, detection of type b (Hib) was performed using real-time PCR targeting the bcsB gene with specific primers previously described by Wroblewski et al. (2013), which are specific for encapsulated type b strains [11]. This amplification was carried out only on samples that tested positive for hpd3.

### Data management and statistical analysis

All data were anonymized and entered into a secure electronic database. Statistical analyses were conducted using IBM SPSS Statistics version 26 (IBM Corp., Armonk, NY). Categorical variables were described using frequencies and percentages. Carriage prevalence for each pathogen was calculated with 95% confidence intervals (CIs). Associations between pathogen carriage and explanatory variables were evaluated using Pearson’s Chi-square or Fisher’s exact test, as appropriate. Variables with p-values < 0.2 in bivariate analysis were included in multivariate logistic regression models to identify independent predictors of carriage. Odds ratios (ORs) with 95% CIs were reported, and statistical significance was set at *p* < 0.05.

## Results

### Characteristics of healthcare workers

A total of 221 paediatric healthcare personnel participated in the study and returned correctly completed questionnaires. The sociodemographic characteristics of participants are summarised in Table 1. The majority were young adults aged 18–35 years (*n* = 156; 70.6%) and female (*n* = 129; 58.4%). Nurses represented the predominant professional group (*n* = 103; 46.6%), and most respondents were employed in paediatrics (*n* = 127; 57.5%).

**Table 1:** Socio-demographics characteristics of the 221 paediatric heathcare workers

Variables (*n* = 221)	Frequency	Percentage (%)
Age 18–35 36–50 ˃ 50 years	156 56 09	70.6 25.3 04.1
Sex Male Female	92 129	41.6 58.4
Occupational group Paediatrician General practitioner Nurse Nursing assistant	09 73 103 36	4.1 33.0 46.6 16.3
Work unit Neonatology Paediatrics Paediatric emergency	65 127 29	29.4 57.5 13.1

### Asymptomatic nasopharyngeal carriage prevalence

Among the 221 nasopharyngeal samples analyzed, real-time PCR detected at least one target pathogen in 81 samples (36.6%). *Streptococcus pneumoniae* (*lytA*-positive) was the most frequently identified bacterium, detected in 54 samples (24.4%). *Haemophilus influenzae* (*hpd3*-positive) was found in 26 samples (11.8%), and *Neisseria meningitidis* (*sodC*-positive) in 22 samples (10.0%) (Table 2). Thirteen participants (5.9%) were co-carriers of two pathogens, and two participants (0.9%) harbored all three organisms (*S. pneumoniae*, *N. meningitidis*, and *H. influenzae*).

**Table 2:** Prevalence of nasopharyngeal carriage of *Streptococcus pneumoniae*, *Neisseria meningitidis*, and *Haemophilus influenzae* among paediatric healthcare workers (*N* = 221)

Pathogens	No. of samples	Positive (*n*)	Prevalence (%)
*Streptococcus pneumoniae*	221	54	24.4%
*Haemophilus influenzae*	221	26	11.8%
*Neisseria meningitidis*	221	22	10.0%

### Distribution of serogroups and types among *Neisseria meningitidis* and *Haemophilus influenzae* isolates

**Fig. 2:**
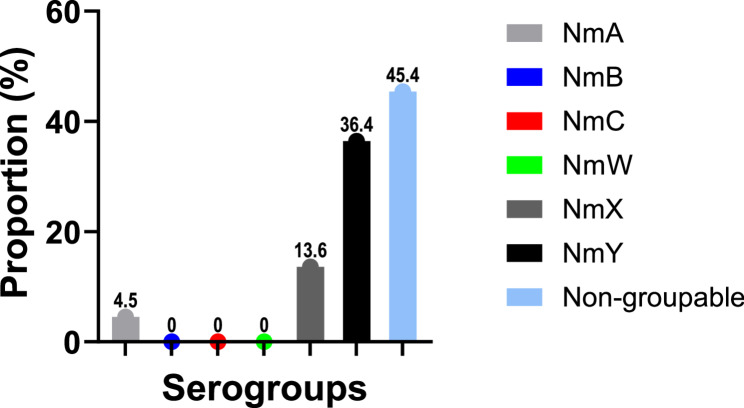
Serogroup distribution of *Neisseria meningitidis* among colonised healthcare workers detected by qPCR typing (*n* = 22). Data are presented as a percentage

Among the 22 *N. meningitidis*–positive nasopharyngeal specimens, multiplex qPCR was performed, revealing marked antigenic diversity (Fig. 2). Serogroup Y was most common (8/22; 36.4%), followed by serogroup X (3/22; 13.6%) and serogroup A (1/22; 4.5%), the latter historically targeted by the MenAfriVac^®^ campaign. Dual serogroup carriage was observed in three participants: two harboured X + Y and one A + Y. Ten samples (45.4%) were negative for all capsular targets tested (A, B, C, W, X, Y), suggesting non-typable, non-encapsulated, or rare serogroups not covered by current vaccines. Among participants carrying serogroupable meningococcal strains (A, X, Y), half were working in hospitals located in northern Benin, within the meningitis belt, while the other half were from southern facilities outside the belt. However, no statistically significant association was observed between the serogroup type and any of the investigated factors, including geographical location, recent RTIs, or vaccination history.

Among the 16 participants who self-reported vaccination against serogroup A *Neisseria meningitidis*, only one tested positive for *N. meningitidis* DNA by real-time PCR. However, this isolate was non-groupable, showing no amplification with any of the tested serogroup-specific primers.

All 26 *H. influenzae* (hpd3-positive) samples were negative for the bcsB gene specific to serotype b (Hib), confirming the absence of Hib colonisation. No other capsular serotypes were targeted by PCR.

### Factors associated with *Streptococcus pneumoniae* colonisation

To identify potential factors associated with *S. pneumoniae* carriage among healthcare workers, we conducted a bivariate analysis using the chi-square test or Fisher’s exact test as appropriate. The variables assessed included: age group, sex, occupational group, work unit, vaccination status, cohabitation with children under 10 years of age, residence in the meningitis belt, and history of recent respiratory tract infection (RTI). Multivariate logistic regression identified three variables statistically associated with *S. pneumoniae* carriage at the 5% significance level (Table 3):

**Table 3:** Factors associated with nasopharyngeal carriage of *Streptococcus pneumoniae* among paediatric healthcare workers

Factors	*n* (%)	*S. pneumoniae*		OR	IC95%	*P* value*
		Positive *n* (%)	Negative *n* (%)			
Age				1.06	[1.02-2.61]	0.016*
18 – 35	156 (70.6)	45 (83.3)	111 (66.5)			
36 – 50	56 (25.3)	9 (16.7)	47 (28.1)			
˃ 50 years	09 (04.1)	0	09 (05.4)			
Sex				0.98	[0.55-1.75]	0.824
Male	92 (41.6)	25 (46.3)	67 (40.1)			
Female	129 (58.4)	29 (53.7)	100 (59.9)			
Occupational group				0.95	[0.50-1.82]	0.281
Paediatrician	09 (04.1)	2 (3.8)	7 (4.2)			
General practitioner	73 (33.0)	20 (37.0)	53 (31.7)			
Nurse	103 (46.6)	22 (40.7)	81 (48.5)			
Nursing assistant	36 (16.3)	10 (18.5)	26 (15.6)			
Work unit				1.04	[0.60-1.80]	0.431
Neonatology	65 (29.4)	15 (27.8)	50 (29.9)			
Paediatrics	127 (57.5)	33 (61.1)	94 (56.3)			
Paediatric emergency	29 (13.1)	6 (11.1)	23 (13.8)			
Vaccination status				1.04	[0.58-1.88]	0.174
Yes	81 (36.7)	21 (38.9)	60 (35.9)			
No	140 (63.3)	33 (61.1)	107 (64.1)			
RTI in last two weeks				1.07	[0.56-2.02]	0.302
Yes	49 (22.2)	13 (24.1)	36 (21.6)			
No	172 (77.8)	41 (75.9)	131 (78.4)			
Staff lives with children under 10 years				0.87	[0.82-3.67]	0.03*
Yes	33 (14.9)	22 (40.7)	11 (6.6)			
No	188 (85.1)	32 (59.3)	156 (93.4)			
Meningitis belt				1.21	[1.05-3.15]	0.017*
Yes	114 (51.6)	37 (68.5)	77 (46.1)			
No	107 (48.4)	17 (31.5)	90 (53.9)			

*P value ​​˂ 0.05 considered statistically significant

- Age group 18–35 years was associated with higher odds of carriage compared to older age groups (OR = 1.06; 95% CI 1.02–2.61; *p* = 0.016).
- Cohabitation with children under 10 years significantly increased the likelihood of carriage (OR = 0.87; 95% CI 0.82–3.67; *p* = 0.030), Although this association reached statistical significance, the confidence interval for the odds ratio included 1 (OR = 0.87; 95% CI 0.82–3.67), warranting cautious interpretation.
- Residing within the meningitis belt was significantly associated with increased carriage (OR = 1.21; 95% CI 1.05–3.15; *p* = 0.017).

No statistically significant associations were found for sex, occupational category, work unit, vaccination status, or recent RTI (all *p* > 0.05).

### Factors associated with *Haemophilus influenzae* Colonisation

Bivariate analysis did not reveal any significant associations between *H. influenzae* carriage and age, sex, occupational group, work unit, cohabitation with children under 10 years, region of residence, or history of recent RTI (all *p* > 0.05) (Table 4). This suggests that *H. influenzae* colonisation among healthcare workers in this setting may not be strongly influenced by these factors.

**Table 4:** Factors associated with nasopharyngeal asymptomatic carriage of *H. influenzae*

Factors	*n* (%)	*H. influenzae*		OR	IC95%	*P* value*
		Positive* n* (%)	Negative *n* (%)			
Age				0.98	[0.25-1.48]	0.733
18 – 35	156 (70.6)	18 (69.2)	138 (70.8)			
36 – 50	56 (25.3)	7 (26.9)	49 (25.1)			
˃ 50 years	09 (04.1)	1 (3.9)	08 (04.1)			
Sex				1.01	[0.34-2.11]	0.836
Male	92 (41.6)	11 (42.3)	81 (41.5)			
Female	129 (58.4)	15 (57.7)	114 (58.5)			
Occupational group				1.97	[0.96-6.17]	0.496
Paediatrician	09 (04.1)	1 (3.8)	8 (4.1)			
General practitioner	73 (33.0)	12 (46.2)	61 (31.3)			
Nurse	103 (46.6)	9 (34.6)	94 (48.2)			
Nursing assistant	36 (16.3)	4 (15.4)	32 (16.4)			
Work unit				0.97	[0.47-4.70]	0.482
Neonatology	65 (29.4)	5 (19.2)	60 (30.8)			
Paediatrics	127 (57.5)	15 (57.7)	112 (57.4)			
Paediatric emergency	29 (13.1)	6 (23.1)	23 (11.8)			
RTI in last two weeks				1.94	[0.22-1.54]	0.330
Yes	49 (22.2)	8 (30.1)	41 (21.0)			
No	172 (77.8)	18 (69.2)	154 (79.0)			
Staff lives with children under 10 years				0.95	[0.90-3.57]	0.377
Yes	33 (14.9)	12 (46.2)	21 (10.8)			
No	188 (85.1)	14 (53.8)	174 (89.2)			
Meningitis belt				1.49	[0.20-1.80]	0.130
Yes	114 (51.6)	15 (57.7)	99 (50.8)			
No	107 (48.4)	11 (42.3)	96 (49.2)			

*P value ​​˂ 0.05 considered statistically significant

### Factors associated with nasopharyngeal colonisation by *Neisseria meningitidis*

Two variables were significantly associated with *N. meningitidis* carriage (Table 5) :

**Table 5:** Factors associated with *Neisseria meningitidis* colonisation

Factors	*n* (%)	*N. meningitidis *		OR	IC95%	*P* value*
		Positive *n* (%)	Negativ *n* (%)			
Age				0.98	[0,07-1,64]	0.619
18 – 35	156 (70.6)	14 (63.6)	142 (71.4)			
36 – 50	56 (25.3)	8 (36.4	48 (24.1)			
˃ 50 years	09 (04.1)	0	09 (04.5)			
Sex				0.98	[0.36-2.73]	0.757
Male	92 (41.6)	10 (45.5	82 (41.2)			
Female	129 (58.4)	12 (54.5)	117 (58.8)			
Occupational group				0.99	[0.03-2.56]	0.890
Paediatrician	09 (04.1)	2 (9.1)	7 (3.5)			
General practitioner	73 (33.0)	7 (31.8)	66 (33.2)			
Nurse	103 (46.6)	8 (36.4)	95 (47.7)			
Nursing assistant	36 (16.3)	5 (22.7)	31 (15.6)			
Work unit				0.95	[0.30-1.21]	0.014*
Neonatology	65 (29.4)	4 (9.1)	61 (30.6)			
Paediatrics	127 (57.5)	15 (31.8)	112 (56.3)			
Paediatric emergency	29 (13.1)	3 (23.1)	26 (13.1)			
Vaccination status				1.98	[1.13-2.44]	0.450
Yes	81 (36.7)	9 (40.9)	72 (36.2)			
No	140 (63.3)	13 (59.1)	127 (63.8)			
RTI in last two weeks				1.07	[0.56-2.29]	0.302
Yes	49 (22.2)	3 (13.6)	46 (23.1)			
No	172 (77.8)	19 (86.4)	153 (76.9)			
Staff lives with children under 10 years				0.99	[0.68-6.34]	0.765
Yes	33 (14.9)	11 (50.0)	22 (11.1)			
No	188 (85.1)	11 (50.0)	177 (88.9)			
Meningitis belt				1.41	[1.36-8.41]	0.007*
Yes	114 (51.6)	114 (51.6)	108 (54.3)			
No	107 (48.4)	16 (72.7)	91 (45.7)			

*P value ​​˂ 0.05 considered statistically significant

- Residence in the meningitis belt was associated with higher odds of carriage (OR = 1.41; 95% CI: 1.36–8.41; *p* = 0.007).
- Working in the paediatrics department was associated with decreased odds of carriage (OR = 0.95; 95% CI: 0.30–1.21; *p* = 0.014). Although this association reached statistical significance, the confidence interval for the odds ratio included 1 (OR = 0.95; 95% CI: 0.30–1.21), warranting cautious interpretation.

These findings highlight potential geographical and occupational factors influencing meningococcal colonisation risk in healthcare settings.

## Discussion

*Streptococcus pneumoniae*,* Neisseria meningitidis*, and *Haemophilus influenzae* are well-established respiratory pathogens that commonly colonise the human nasopharynx, acting as reservoirs for transmission and potential sources of invasive disease [12, 13]. The present study investigated the asymptomatic nasopharyngeal carriage of these pathogens among paediatric healthcare workers (HCWs) in Benin. To our knowledge, this is the first investigation conducted in a paediatric healthcare setting within the African meningitis belt aimed at assessing occupational exposure risk.

The overall carriage prevalence was 36.6%, with *S. pneumoniae* being the most frequently detected pathogen (24.4%), followed by *H. influenzae* (11.8%) and *N. meningitidis* (10.0%). Two key findings emerged from our molecular analyses. Firstly, capsular typing of *N. meningitidis*-positive samples revealed substantial antigenic diversity. Serogroup Y was the most frequently identified, followed by the unexpected detection of serogroup X, and a single case of serogroup A, historically the primary target of the MenAfriVac^®^ campaign [14]. Notably, over one-third of the *N. meningitidis*-positive samples were non-groupable using conventional PCR primers targeting serogroups A, B, C, W, X, and Y, suggesting the presence of either non-encapsulated strains or less common serogroups. Secondly, none of the *H. influenzae* strains identified in this study tested positive for the *bcsB* gene specific to serotype b (Hib), indicating that only non-type b or non-encapsulated strains were circulating among the study population.

The *S. pneumoniae* carriage rate observed (24.4%) is considerably higher than those reported in similar populations from high-income countries. For instance, Almeida et al. (2020) in Portugal reported a prevalence of 14.8% among adult HCWs [15], while Steurer et al. (2022) in Austria recorded a much lower rate of 3.7%, using comparable methods [16]. Variations in geographic context, vaccination policies, diagnostic methodologies, and population characteristics may contribute to these discrepancies. By comparison, a recent longitudinal study in the United States by Waghela et al. (2025), which employed saliva samples from healthcare personnel with suspected COVID-19, reported a cumulative individual *S. pneumoniae* carriage prevalence of 21% over a four-month period, with between one and ten samples collected per participant [17]. Although our study used a single-point sampling approach, which may underestimate transient carriage, our results still revealed a higher point prevalence. This may reflect a heightened baseline exposure risk among paediatric healthcare workers in the meningitis belt, where frequent contact with unvaccinated children and persistent colonisation are more likely.

In the absence of local baseline data on meningococcal carriage in Benin, we referred to a study conducted in Mali, a neighbouring country within the African meningitis belt, which reported a 9.1% carriage prevalence among adults aged 15–29 years (Basta et al., 2012) [18]. This is consistent with the 10% prevalence observed in our study. In contrast, a much lower prevalence of 1.14% was reported among Austrian healthcare workers by Steurer et al. (2020) [19]. Furthermore, while our molecular analyses identified serogroups Y and X as predominant among carriers, contrasting with the predominance of genogroup B reported in the Austrian study, it is worth noting that most participants in our study who reported being vaccinated against meningitis specifically referred to immunisation against serogroup A during mass vaccination campaigns conducted in response to recent outbreaks. The observed prominence of non-A serogroups in our study may be influenced by shifts in meningococcal epidemiology across the African meningitis belt, where mass immunization campaigns have primarily targeted serogroup A. The identification of serogroup X among healthcare workers is noteworthy, given its implication in recent outbreaks in neighbouring countries such as Ghana, where it accounted for over a quarter of confirmed meningitis cases in 2020. This reinforces the need for close surveillance and preventive strategies targeting emerging serogroups [20]. Given that HCWs in Benin are primarily immunised against serogroup A during mass campaigns, the emergence of other serogroups likely reflects insufficient immunological coverage, underscoring the need for broader-spectrum meningococcal vaccines. Although no statistically significant association was found between geographic origin and specific serogroups in our study, it is noteworthy that serogroupable isolates (A, X, Y) were evenly distributed between healthcare workers from within and outside the meningitis belt. This may suggest a broader circulation of these serogroups beyond traditional endemic zones, highlighting the importance of expanded vaccine coverage.

The nasopharyngeal *Haemophilus influenzae* carriage prevalence (11.8%), aligns with data from South Asia. Amritha G.N et al. (2020) reported an 11% prevalence among Indian HCWs [21], while Subramanya et al. (2016) documented a 15% rate among Nepali HCWs, higher than the 8% observed in the general population [22]. These findings support the hypothesis that occupational exposure, particularly in paediatric settings with frequent and close contact with unvaccinated or partially immunised children, may increase the risk of asymptomatic colonisation among staff. This is in line with previous evidence showing that close contact with children who often exhibit high nasopharyngeal and oropharyngeal carriage rates contributes to increased colonisation in adults. It is important to note, however, that while Hib vaccination has reduced invasive disease due to *H. influenzae* type b, it does not affect the carriage of nontypeable *H. influenzae* or other encapsulated types (a, c, d, e, f), which may still circulate widely [23]. Consistent with global trends, our study did not identify serotype b strains, reinforcing the clinical relevance of nontypeable *H. influenzae* (NTHi). Although generally considered less invasive than Hib, NTHi strains are increasingly recognised as significant contributors to respiratory tract infections, particularly in vulnerable populations [24]. Our findings underscore the importance of sustained surveillance and the development of tailored preventive strategies in healthcare settings. In regions where infant Hib vaccination has reduced the circulation of type b strains, non-type b or unencapsulated variants may persist and pose ongoing risks. Asymptomatic carriage among healthcare workers remains a major concern due to the potential for onward transmission to high-risk paediatric patients, especially in resource-limited environments where both vaccination coverage and infection control practices may be suboptimal [25].

In our study, residence within the African meningitis belt was significantly associated with asymptomatic carriage of both *Streptococcus pneumoniae* and *Neisseria meningitidis*, suggesting a potential geographical influence on colonisation dynamics. This observation is consistent with previous research indicating that environmental and geographic factors characteristic of the meningitis belt contribute to elevated carriage rates and a higher risk of invasive disease [9, 25]. For instance, Mueller et al. (2012) in Burkina Faso reported seasonal hyperendemicity of both pneumococcal and meningococcal meningitis, particularly during the dry season [9]. These environmental conditions are thought to compromise the integrity of the nasopharyngeal mucosa, facilitating bacterial colonisation and subsequent invasion [26]. Beyond geographic location, additional factor were significantly associated with asymptomatic carriage in our cohort. Young age (18–35 years) emerged as a significant risk factor for *S. pneumoniae* colonisation, a finding that may reflect increased interpersonal contact and social mobility typically observed in this age group. These results are consistent with prior studies that have identified age and household contact with children as key determinants of pneumococcal colonisation. For instance, Steurer et al. (2022) reported that living with children under the age of 8 and being aged 36–45 years were independently associated with *S. pneumoniae* carriage, with prevalence rates of 11.6% and 6.7%, respectively [16]. Interpersonal contact likely increase exposure to paediatric reservoirs. Other studies have similarly identified occupational factors. In Iran, Samadpanah et al. (2020) observed higher colonisation rates among nurses and interns and noted a significant association with smoking status [26]. Similarly, Subramanya et al. (2016) reported that resident doctors and smokers had higher odds of carriage [22]. Although some associations in our analysis were statistically significant based on p-values, the corresponding confidence intervals included 1.00, suggesting caution in interpretation. For example, working in paediatrics was associated with lower odds of meningococcal carriage (OR = 0.95; 95% CI: 0.30–1.21; *p* = 0.014), and living with children under 10 appeared to increase pneumococcal carriage (OR = 0.87; 95% CI: 0.82–3.67; *p* = 0.030). These findings may indicate potential associations, but wide confidence intervals overlapping 1.00 raise the possibility of chance findings. Additionally, the study did not investigate smoking habits, the increased carriage observed among younger staff may reflect behavioural or environmental exposures not fully captured in the current analysis.

In contrast, our data did not identify any significant association between *Haemophilus influenzae* carriage and the sociodemographic or professional variables assessed. This finding is consistent with the results of Amritha G.N. et al. (2020), who reported no significant correlations between demographic factors and *H. influenzae* colonisation in healthcare settings [21]. The relatively uniform distribution of carriage across subgroups in our population may point to broader environmental or institutional factors influencing transmission.

In summary, our findings highlight the important role of age, geographic location, and childcare contact in shaping asymptomatic *S. pneumoniae* and *N. meningitidis* carriage among HCWs. The variability in carriage patterns observed across settings and pathogens highlights the need for context-specific epidemiological data to inform effective infection prevention and control (IPC) measures. In resource-limited settings, where immunisation coverage among healthcare workers is often incomplete and IPC infrastructure may be suboptimal, systematic screening and targeted interventions could play a crucial role in mitigating the risk of transmission to vulnerable paediatric populations. Future research should adopt longitudinal approaches to better capture the temporal dynamics of asymptomatic carriage and antimicrobial resistance, and further explore the interplay of behavioural, occupational, and environmental risk factors shaping colonisation in healthcare environments.

### Strengths and limitations

This study provides valuable insights into the asymptomatic carriage of key bacterial pathogens among paediatric healthcare personnel in a region within the African meningitis belt, a population group that has been understudied to date. The use of sensitive molecular techniques such as qPCR allowed for accurate detection and serotyping of *Streptococcus pneumoniae*, *Neisseria meningitidis*, and *Haemophilus influenzae*, contributing to a detailed understanding of colonisation patterns and potential risks in this high-exposure setting.

However, there are limitations to consider. The absence of a control group consisting of non-healthcare workers limits the ability to directly assess whether healthcare personnel are at increased risk of carriage compared to the general population. Additionally, the cross-sectional design and single time-point sampling restrict the understanding of temporal dynamics and duration of bacterial carriage. Another limitation of this study is the absence of data on participants smoking habits, which could potentially influence nasopharyngeal colonisation. Furthermore, vaccination status was based on self-report and was not confirmed through vaccination cards, which may have introduced recall or reporting bias. Future longitudinal studies including a broader comparator population would provide more robust evidence regarding the occupational risk of carriage among healthcare workers. Furthermore, the identification of *Streptococcus pneumoniae* in this study relied solely on the detection of the *lytA* gene via real-time PCR. While *lytA* is widely recognized as a reliable marker for pneumococcal detection, it is important to note that homologous sequences have been identified in other *Streptococcus* species, such as *S. mitis* and *S. oralis*. This genetic similarity can lead to false-positive results, particularly in samples from the oropharyngeal region, where these commensal species are prevalent. To enhance specificity and reduce the risk of misidentification, incorporating additional pneumococcus-specific targets, such as the *piaB* gene, is recommended. The combined detection of *lytA* and *piaB* has been shown to improve the accuracy of pneumococcal identification in molecular assays [27].

## Conclusion

*N. meningitidis*, *H. influenzae* and *S. pneumoniae* are important causes of meningitis in paediatric hospitals in Benin. This study highlights a notable prevalence of asymptomatic nasopharyngeal carriage of *Streptococcus pneumoniae*, *Neisseria meningitidis*, and *Haemophilus influenzae* among paediatric healthcare workers in Benin. The findings underscore the diversity of circulating strains, including non-typeable and non-vaccine serogroups, which may pose a risk to unvaccinated or immunocompromised paediatric patients. The predominance of non-type b *H. influenzae* and the detection of serogroups not targeted by routine adult vaccination policies emphasise the need for ongoing surveillance and consideration of targeted prevention strategies. Strengthening infection control practices and evaluating the need for broader immunisation coverage among healthcare workers could play a critical role in reducing transmission within hospital settings.

## Supplementary Information

Supplementary Material 1.

## Data Availability

The datasets generated and/or analysed during the current study are available from the corresponding author on reasonable request.

## Abbreviations

HCW: Health care worker
HCWs: Health care workers
PCR: Polymerase chain reaction
N. meningitidis: Neisseria meningitidis
H. influenzae: Haemophilus influenzae
S. pneumoniae: Streptococcus pneumoniae
RT-PCR: Real-time polymerase chain reaction
qPCR: Quantitative Polymerase Chain Reaction
Nm: Neisseria meningitidis
Spn: Streptococcus pneumoniae
Hi: Haemophilus influenzae
LytA: Autolysin gene specific to Streptococcus pneumoniae
SodC: Superoxide dismutase gene specific to Neisseria meningitidis
Hpd3: Protein D gene specific to Haemophilus influenzae
Hib: Haemophilus influenzae type b
OR: Odds Ratio
CI: Confidence Interval
MenAfriVac®: Meningococcal A conjugate vaccine used in Africa
IPC: Infection prevention and control
RTI: Respiratory tract infection
