# Supplementary material for: Nasopharyngeal carriage and risk factors of major meningitis pathogens among asymptomatic healthcare workers in paediatric units in Benin, with serogroup distribution of Neisseria meningitidis
Source: BMC Infect Dis. 2025 Aug 22;25:1056. doi: 10.1186/s12879-025-11492-3 (PMC12372238; doi:10.1186/s12879-025-11492-3)
Supplement: Supplementary file 1 — Supplementary Material 1. [file 12879_2025_11492_MOESM1_ESM.pdf]

RESEARCH TITLE: Epidemiological profile and characterisation of meningitis-associated pathogenic bacteria isolated from healthcare workers in paediatric units.

Principal investigator: Chakir Ishola BELLO

Study Period: September 2023 – December 2024

Confidential – For research purposes only

QUESTIONNAIRE

Date ………/………./202 **Participant ID Code :**………………………….………..………………….

A 1. Department :……………………….……… **A 2.** Hospital : ……………......……………………………

Q 1. Sex : Male /___/ Female /___/ **Q 2.** Age : /_____/ years

Q 3. Occupational group /____/ **:** **1**= Specialist ; **2**= General practitioner ; **3**= Nurse ; **4**= Nursing assistant

Q 4. Work unit /___/ **:** **1**= Neonatology ; **2** = Paediatrics ; **3** = Paediatric emergency

Q 5. Have you experienced respiratory tract infection symptoms in the past two weeks? /___/

1= Yes ; **2**= No

Q 6. Do you live with any children under 10 years of age? /___/ : **1**= Yes ; **2**= No

Q 7. Have you been vaccinated against meningitis? /____/ : **1**= Yes ; **2**= I don't know ; **3**= No

If **yes,** specify:

Less than 1 year ago ☐ ; 1–2 years ago ☐ ; 2–5 years ago ☐ ;

Over 5 years ago ☐ ; Over 10 years ☐
